# Female managers’ organizational leadership during telework: experiences of job demands, control and support

**DOI:** 10.3389/fpsyg.2024.1335749

**Published:** 2024-05-30

**Authors:** Ola Nordhall, Raman Kaur, Linnea Törnblom, Igor Knez

**Affiliations:** Faculty of Health and Occupational Studies, Department of Occupational Health, Psychology and Sports Sciences, University of Gävle, Gävle, Sweden

**Keywords:** female managers, telework, demands control support, interviews, theory-driven thematic analysis

## Abstract

The frequent use of telework during the COVID -19 pandemic has created a more challenging work situation for managers who need to lead effectively in the virtual space, this especially concerns female managers. Therefor it is of importance to investigate female managers’ experiences of job related demands, control and support within this work context. Accordingly, we investigated female managers’ experiences of demands, control and support in their organizational leadership during telework. The present study used a deductive, theory-driven, qualitative approach with predetermined themes defined within the demand-control-support model. Data were collected by semi-structured interviews. The female managers had at least 50% of their working hours as telework. The results showed that the female managers experienced *demands* in terms of hard, fast or even excessive work in order to be available and solve complex problems, and *control* as varied work content, new learning, planning and decision freedom. *Support* was experienced in terms of attentive superior manager, good cooperation and helpfulness among colleagues. Theoretical and practical implications of the results are discussed in terms of telework situation for female managers.

## Introduction

The frequent use of telework during the COVID -19 pandemic has created a more challenging work situation for managers who are supposed to lead employees from a homebound work context (Plotnikof et al., 2020). Also, in work organizations there are requirements for managers to be available, develop, perform, change and meet needs of both employees and business. This especially concerns first-line managers who play important roles of interfacing with employees and reporting to superior (senior) managers about the day-to-day operations of an organization (Hales, 2006; Ellström and Ellström, 2018). In addition, a manager needs to solve complex problems, make difficult decisions and act as a sounding board and support toward his/her employees. Accordingly, it is important that managers themselves receive support. In the private, compared to the public, sector they have stronger support in administrative tasks. The lesser support in the public sector is associated with higher sickness rates (Berntson et al., 2012). Also, if a manager experiences high demands and low support during telework, it may lead him/her the to act in an undesirable way that employees may be affected by Serviss (2021). Moreover, communication technology demands that managers shift to “digital leadership” creating new challenges for managers in their leadership position (Gfrerer et al., 2020).

A manager is part of- and affected by the organizational framework just like the rest of the employees. During the COVID −19 pandemic, telework escalated and became a mandatory work context for many employees and managers. Of those working in the EU, almost 40% started to telework full-time due to the pandemic [see Athanasiadou and Theriou (2021)]. Telework means that all or part of the occupational work is done from home/ another location via communication technology, such as computers or telephones [see Lindström et al. (1997), Allen et al. (2015), and Athanasiadou and Theriou (2021)]. During the COVID −19 pandemic telework became more or less synonymous with homebound work (Widar, 2022; Widar et al., 2022).

With telework leadership has met new challenges in organizations where managers need to learn how to lead effectively in the virtual space (Contreras et al., 2020; Korkeakunnas et al., 2023). During telework, a manager needs to find new methods to be active in communication, supervisory skills, create motivation, participation, create social context, form clear goals and a vision. This may lead to difficulties in delegating decisions related to challenges in creating trust and good relationships in the virtual space (Benjamin and O’Reilly, 2011; Cortellazzo et al., 2019; Widar et al., 2022). This, in turn, may increase the manager’s stress and reduce his/her tolerance for uncertainties (Langfred and Rockmann, 2016; Yarberry and Sims, 2021).

While some studies indicate gender differences in leadership behavior (Shen and Joseph 2021), gender differences in work conditions for teleworking managers seems to be a rather sparsely researched area, where a few studies have investigated gender specific perceptions of work conditions in relation to family conditions and mental health outcomes [see Xie et al. (2021) and Rahnfeld et al. (2023)].

Furthermore, women have low representation in management positions in the Western world. Women’s participation in the workforce has however increased in lower and medium level positions, even though women are still poorly represented in higher positions (Velte, 2017). Accordingly, it is important to investigate female managers’ experiences of job demands, control and support during telework.

### The JDCS model

One of the most influential models of psychosocial work conditions is the.

job demands-control-support model (JDCS model) by Karasek and Theorell (1990). The JDCS model is the conceptual frame of reference of the present study. In line with that, a theory driven deductive approach was implemented in formulating the interview guide and research questions and analyzing the qualitative data of the present study.

Job demands are described as “how hard you work” and include deadlines, how many work tasks must be completed in a week and how much is done in an hour (Karasek and Theorell, 1990). Work that is demanding can still give job satisfaction if the employee has opportunities for greater decision-making (Karasek, 1979). According to the JDCS model, the following constitutes job demands, or its opposite: work fast, work hard, excessive work, enough time, conflicting demands, intense concentration, task interrupted, hectic job and wait on others. These work characteristics involve mental attention and stimulation of the individual elicited by work tasks and workload (Karasek et al., 1998; Sanne et al., 2005).

Job control is the ability to control one’s own activities and skills, that is “skill discretion” which provides more stimulation to overcome difficulties and reduces stress (Karasek and Theorell, 1990; Paulsson et al., 2005). Gaining influence means an opportunity for more self-determined decisions. This type of “decision authority” influences one’s own work situation and that work tasks are carried out. Combinations of “skill discretion” and “decision authority” make up “decision latitude,” equaling control which may increase work engagement and decrease turnover intentions (Karasek and Theorell, 1990; McKenna and Jeske, 2021). Control may also manifest itself as autonomy experiences (self-governance) which may increase job satisfaction and productivity (Langfred, 2013). When employees are not given the opportunity for autonomy in their work, and also have high demands, there is a negative effect on mental health and greater risks of various diseases (Karasek and Theorell, 1990; Too et al., 2020). According to the JDCS model, the following constitutes control, or its opposite: learn new things, repetitive work, creativity, high skill level, variety, develop own abilities, allows your own decisions, little decision freedom, a lot of say and education required by job (Karasek et al., 1998; Sanne et al., 2005).

Job support refers to “social support,” which applies to helpfulness and availability from co-workers and managers (Johnson and Hall, 1988; Karasek and Theorell, 1990). Social interaction and support between employees and between employees and managers are of importance for job satisfaction, health and good behavioral reactions. Employees who have low social support at work often find it difficult to achieve a good work-life balance and may show emotional fatigue (Karasek and Theorell, 1990; Umene-Nakano et al., 2013). However, if employees receive social support at the workplace it increases their creativity and new knowledge (Karasek and Theorell, 1990; Op den Kamp et al., 2020). According to the JDCS model, the following constitutes support, or its opposite: superior manager concerned, helpful supervisor, superior manager pays attention, hostile supervisor, superior good organizer, coworkers competent, coworkers interested in me, hostile coworkers, friendly coworkers, coworkers work together and helpful coworkers (Karasek et al., 1998; Sanne et al., 2005).

The JDCS model predicts that job demands, work variety and decision freedom (i.e., control) and social support (from colleagues and supervisors) interact in affecting mental health. Strong job demands may have a less negative effect in employees who gain autonomy and social support in their job, i.e., job control and support may buffer the negative impact of stressful job demands (Karasek and Theorell, 1990; Rubino et al., 2012; Treiber and Davis, 2012). A balance between work-related demands, control and social support may increase the opportunities of avoiding, or reducing, negative effects of stress and adverse psychosocial work conditions on health (Karasek and Theorell, 1990).

This model is one of the most widely used theoretical frameworks that relate job characteristics to stress, health, well-being and sustainability for employees and organizations. It has been used to explain a wide range of organizational phenomena, such as stress, engagement, burnout, emotional exhaustion, work-life balance, job-related performance, satisfaction and anxiety as well as bullying [see Chirico (2016), Fila et al. (2017), Asif et al. (2018), Matilu and K’Obonyo (2018), and Finstad et al. (2019)]. However, the interactive predictors have faced contradictory findings and moderating hypotheses have received modest support [see Ibrahim and Ohtsuka (2014)].

### Managers, telework and gender

One challenge for managers during telework is to maintain high productivity in employees. To achieve this, leadership must be efficient, and the manager must possess good self-leadership (Nelson, 2000). Managers who do not employ good self-leadership (stronger self-control) may find it more difficult to lead others in a sustainably way (Forgas et al., 2009).

Having social support during telework by good relationships with co-workers enables managers to use and develop skills and knowledge. If the manager has an opportunity for more decision-making during telework, he/she is better able to allocate own time and become more effective. This gives more potential to find a balance between occupational work and private life, for example, by time for exercise, achieving and completing goals, as well as for improving self-discipline (Benjamin and O’Reilly, 2011).

However, telework may increase social isolation, that in turn may increase the stress level and adverse mental health in general (Palm et al., 2020; Carillo et al., 2021; Kniffin et al., 2021). According to Wang et al. (2021), social support is important to counteract adverse mental health when working remotely as a result of the Covid-19 pandemic. Furthermore, Shahidul (2013) indicates that social support and social interaction are important for new employees to become part of the team, as well as to promote community in the work group.

It has been shown that those who frequently vs. rarely telework report more stress as related to indistinct organization, conflicts and individual job demands (Heiden et al., 2021).

In view of the above, it is of value to gain more knowledge of how individual managers experience their leadership during telework.

Shen and Joseph (2021) have reported metanalytical gender differences in some sub-categorizes of leadership emergence, effectiveness, behavior and motivation but not in other sub-categorizes of these leadership outcomes. Accordingly, female and male managers may differ in some respects regarding leadership performance and experiences.

During telework, private life and the workplace boundaries tend to dissolve. This has proven particularly distinctive for women compared to men. Women compared to men are expected to combine several roles in the private sphere because they are expected to carry out chores that belong to the home when working remotely (Mann and Holdsworth, 2003). Also, teleworking women compared to men report more stress and fatigue (Heiden et al., 2021).

#### Female managers

Women vs. men tend to have a disadvantage in psychosocial work conditions in terms of higher job demands and lower job control (Sekine et al., 2011). In addition, women have been shown to be more affected by job demands and psychological distress and perceive job demands as job-family demands and job support as job-family support (Banerjee and Doshi, 2020; Xie et al., 2021). Also, for females, job demands predict work–family conflicts and exhaustion and work–family conflict predicts lack of well-being (Akram, 2020; Gu et al., 2020; Rahnfeld et al., 2023). Other studies have shown mixed results regarding gender differences in the association between JDCS variables, work–family conflict and well-being. For example, work–family conflict has been shown to mediate the relation between job demands and support and job satisfaction for males and females. However, such a mediating role of work–family conflict were only found for female employees when job control was the predictor and job control was negatively related to work–family conflicts only in female employees [see Hwang and Ramadoss (2017)]. In addition, women in management positions may face difficulties due to gender stereotypes and prejudices and may therefore experience different demands compared to men in manager roles (Schein, 2007).

Regarding the above, it is of importance to understand how individual female managers experience their leadership in terms of work conditions.

According to the JDCS model, an individual may be in a passive, active or iso-tense state that affects his/her health. The passive state implies low job control in combination with low job demands. The iso-tense situation is characterized by the individual having high demands, low control and low support. An iso-tense state is an unhealthy state, as the individual is exposed to a high risk of negative stress and thus adverse mental health (Karasek and Theorell, 1990).

Due to their telework, which is frequently characterized by high demands, low control and low social support in their occupational work, managers may easily end up in the iso-tense state which may be of disadvantage for their mental health. However, if managers experience a balance between work-related demands, control and social support, where all of these three factors are quite strong, they may be in an active state which is more optimal for their mental health [see Karasek et al. (1998) and Sanne et al. (2005)].

To our knowledge just a few studies have investigated these types of organizational phenomena and none of these studies included individually experiences of teleworking female managers. Accordingly, there is a need to gain more specific knowledge and understand how individual female managers experience their leadership in terms of job-related demands, control and support as defined within the framework of the JDCS model during telework.

### Aim and research questions

Given the above and that female managers’ experiences of psychosocial work conditions in their organizational leadership during telework has seldom been addressed by previous studies [see Xie et al. (2021), Korkeakunnas et al. (2023), and Rahnfeld et al. (2023)], our aim was to investigate female managers’ experiences of job demands, control and support during telework. More precisely, how do female managers experience:

- Job demands in terms of *work fast, working hard, excessive work,* and *conflicting demands* in their organizational leadership during telework?
- Job control in terms of *learn new things, creativity,* var*iety* and *decision freedom* in their organizational leadership during telework?
- And job support in terms of *superior manager concerned, helpful superior manager, helpful coworkers,* and *coworkers work together* in their organizational leadership during telework?

Since our theoretical frame of reference is the JDCS model (Karasek and Theorell, 1990), we used a deductive, theory-driven, approach. Consequently, the interview guide, research questions and the analyses were based on, by the model defined, predetermined themes [see Azungah (2018)]. Accordingly, in contrast to past quantitative studies applying the JDCS model [for overviews see, e.g., Chirico (2016), Fila et al. (2017), Asif et al. (2018), and Matilu and K’Obonyo (2018)], the present study captured individual experiences expressed as qualitative data. In contrast to past inductive qualitative studies on management, telework and/or the JDCS model [see, e.g., Widar et al. (2021), Kelly et al. (2022), and Ricciardelli and Carleton (2022)], for a deductive exception, [see Fernemark et al. (2020)], the female managers’ experiences were studied within the framework of pre-determined themes deductively derived from a well-supported occupational psychology theory of job demands, control and support. This means that the theoretically defined themes were given an experiential ideographic content, elaborating the understanding of female managers’ experiences of job demands, control and support during telework.

## Method

Based on Karasek and Theorells’ (1990) job demands-control-support model (JDCS model), three pre-determined main themes of demands, control and support, including four sub-themes each, were applied. Each sub-theme was reformulated into questions, defining the semi-structured interview guide; see the Materials section for detailed descriptions.

### Participants

The participants consisted of seven female managers (out of ten participants, response rate = 70%) working within the same type of Swedish public sector, stationed at different geographical locations in Sweden (regarding saturation and credibility see “Procedure” and “Trustworthiness in the present study” below). The participants were first-line managers in an organization (a state agency) that secures property ownership and make geodata available to society. All participants had prior organizational leadership experiences and at least 50% of their working time was telework. They were between 38 and 61 years old (mean age = 51.14, *SD* = 8.00), and their employment time in the organization at hand varied from 1 to 22 years (mean employment time = 12.14 years, *SD* = 7.78). Their current manager-position experiences varied between 1 and 14 years (mean manager-position time = 6.86 years, *SD* = 5.10). The participants had different levels of education; 45% high school education, 45% academic education and 10% other types of education. All participants had the following manager work tasks: work environment-, personnel-, leading, delegating, result-, budget- and operational responsibility, as well as reporting to superior (senior) managers about the day-to-day operations of the organization.

### Materials

A semi-structured interview guide was designed based on “The Job Content Questionnaire” (JCQ) (Karasek, 1985; Karasek et al., 1998; Sanne et al., 2005), with follow-up questions (see Supplementary Appendix 1). By this, a theoretical framework and increased validity and reliability of the interview guide were obtained. The main theme (bold headings) including sub-themes (italicized) were as follows:

#### Job demands

The following sub-themes were chosen (Karasek, 1985; Karasek et al., 1998): *Work fast* (how quickly work tasks are completed); *Work hard* (amount of workload and effort); *Excessive work* (commitment and dedication to work); *Conflicting demands* (how managers prioritize and solve several work tasks that have to be done at the same time).

#### Job control

The following sub-themes were chosen (Karasek, 1985; Karasek et al., 1998): *Learn new things* (opportunity for learning at the work); *Creativity* (creativity in the work tasks); Var*iety* (changes that the manager can make based on the working day/time and tasks); *Decision freedom* (opportunity to make own decisions).

#### Support

The following sub-themes were chosen (Karasek, 1985; Karasek et al., 1998): *Superior manager concerned* (interest in, and concern for, the individual female manager’s work); *Helpful superior manager* (emotional and/or practical support for the individual female manager); *Helpful* c*oworkers* (giving support to each other and how accommodating managers are to each other at work); *Coworkers work together* (collaboration).

### Procedure

We contacted a senior manager responsible for operations in the current area who e-mailed us information of 10 female managers interested in participating in the present study. We e-mailed a covering letter to them and suggested interviews They gave their written consent to participate and received an interview appointment. We interviewed seven participants and by this number of participants we obtained a richness of information in that the participants had different manager experiences, were of different ages and genders and had different organizational positions and employment times, see below. Compared to other participants, answers of the 6th and 7th interview were similar in content. This indicates that saturation was obtained for these interviews. Accordingly, we did not conduct further interviews [see Hennick et al. (2017), for a critical discussion of saturation in terms of number of participants, see O’Reilly and Parker (2013) and Thorne (2020)].

Restrictions regarding external visitors to the organization due to the COVID-19 pandemic meant that all the interviews were carried out remotely. The interviews were conducted during April 2022.

At the time of the interviews, and in accordance with the covering letter, the participants received information about the aim of the study, estimated time consumption and a request if they approved a recording of the interview. They also had the opportunity to ask questions about the study prior to the interview. After consent from the participants (see below under “Research ethics”), the semi-structured interviews were started, recorded via “Zoom video recording” and with the app “Voice record.” Two of the authors conducted the interviews. During the interviews, only the participant and interviewers were present in the digital “room.” This was to allow participants to speak freely.

One semi-structured interview was conducted with each participant. The duration of the interviews was between 28 and 77 min. Before we ended the interview the participant was given an opportunity to ask questions and add information.

### Data analyses

We conducted a deductive, theory-driven, thematic analysis in line with the Hayes (1997) model, see also Hayes (2000), involving four steps [see Azungah (2018) for a similar approach]: First, the theoretical-predetermined main- and sub-themes were formulated. Second, the interview guide was designed based on the main- and sub-themes, interviews were conducted and transcribed. Data were prepared in the form of transcripts. Third, each theme was analyzed separately, determining which data belonged to each theme. The transcripts were coded by identifying key concepts as initial coding categories, in accordance with the JDCS model. The process was repeated once again to ensure that all relevant data were properly coded and sorted under the correct theme. Fourth, the text for each theme was read separately in order to check that the respondents’ answers were properly analyzed. Finally, we summarized all descriptions under each theme, regardless of who said what, and representative quotes from the transcripts that were considered to reflect adequately the summaries for each theme were selected.

### Research ethics

Since no sensitive personal data were collected, such as the health of the participants, no formal ethical approval was needed for the present study in accordance with Swedish juridical restrictions concerning research ethics [see World Medical Association (n.d.)]. The principles of the Declaration of Helsinki [see World Medical Association (n.d.)]; and APA (American Psychological Association, 2020) were carefully followed regarding informed consent from the participants, treatment of participants and handling of research data. All participants gave their informed written consent and were anonymous with reference to confidentiality requirements. They were all assured that their individual answers could not be linked to a specific person. Finally, all participants received information about the recording purpose, and they were asked if they agreed to the interviews being recorded.

### Trustworthiness

In terms of credibility, dependability, and transferability, trustworthiness has been met in the following ways [see Graneheim and Lundman (2004) and Rapp et al. (2021)]:

*Credibility.* Job demands, control and support may be of different types and might be experienced in different ways at different offices (see above). This in turn may be explained by different job characteristics which prevail in the various offices [see De Jonge and Kompier (1997)]. By including female managers from offices stationed at different geographical locations in Sweden, we obtained different work perspectives, experiences and job characteristics, which is of relevance to obtain richness of information [see Rashid et al. (2004)]. Additionally, participants of different ages, educational background and employment- and position times within the present organization were included in the present study. This further contributed to a greater variation in experiences of the current phenomena, thereby strengthening the credibility of the present study.

*Dependability* was obtained by high level of agreement between the researchers of the present study concerning all steps of the data analysis. Initially, we asked the same questions during all interviews. First, coding and determination of data belonging to each theme, respectively, were done independently by two authors. Transcripts were coded by identifying key concepts as initial coding categories, in accordance with the JDCS model. Second, an open dialog and joint secondary coding between the authors contributed to consistent judgments and interpretations of the results obtained. This showed a strong agreement between the authors, suggesting a strong interrater reliability.

*Transferability* was obtained by consistently applying the well-supported theoretical framework of the JDCS model, as well as by providing the specific organizational context, detailed descriptions of the pre-determined themes (and sub-themes), and quotes from respondents for each sub-theme. Also, we demonstrated that the respondents’ individual experiences of the job demands, control and support (sub-themes included) are in line with the theoretical accounts of these processes.

## Results

Participants’ answers are reported in line with the three predetermined main themes of demands, control and support (bold), each including four sub-themes (italicized) describing in detail the respondents’ experiences of telework management related to job demands, control and support. To promote confidentiality, the seven respondents are referred to as R1-R7. See Table 1 for a summary of the results obtained.

**Table 1:** Main experiences that emerged in the female managers’ answers related to the pre-determined theory-driven main themes (bold) and sub-themes (italics) during telework.

Demands	Control	Support
*Work fast:* -Less interrupted by others. -Direct response by digital chat. -Be quick and flexible. -Handle changes in the team. -Lack of face-to-face dialogue. *Work hard:* -Engaged and energizing. -Calm and stress-resistant. -Push oneself and others. -Increased effort in work. -Many meetings. -Decreased availability to others. *Excessive work:* -Work-environmental commitment. -Being the “spider in the web.” -Handle complex personnel and organizational tasks. -Working more during telework. -Digital meetings. *Conflicting demands:* -Multitasking. -More efficient during telework. -Prioritizing personnel and operational issues. -Do remaining tasks in evenings and weekends.	*Learn new things*: -When new/unusual things happened. -By educational programs. -By own information search. -Lost “overhearing” during telework. -By digital tools. *Creativity*: -Finding alternative solutions. -Activity for teams. -By physical presence in the office. -Hindered during telework (monotonous information meetings). Var*iety*: -Handle diverse subjects. -More variation in personal work tasks. -Telework facilitates organizational collaboration. -Less variation by working from home during telework. *Decision freedom*: -Not in general affected by teleworking. -Within certain frameworks. -Delegating tasks to employees. -By weekly reconciliations. -Frequent decision checking during teleworking delays decisions.	*Superior manager concerned:* -Highlighting good work and suggestions for improvement. -Feedback via coordination meetings. -Easier asking questions and getting feedback in the office. -Superior managers’ attention and concern the same during telework. *Helpful superior manager:* -Due to mutual dependence. -By their commitment, weekly reconciliations, support and feedback. -By prioritizing availability and support during telework. *Helpful coworkers:* -Facing the same problems. -Solve problems together. -Showing concern (praise and confirmation). -Suggestions for improvement. -Act as sounding board. -Helpfulness not affected by the physical distance. *Coworkers work together:* -Consensus that colleagues cooperate. -For the work to flow. -By asking for help, joint planning and helping. -Digital tools facilitate getting in touch. -Telework does not affect the cooperation.

### Demands

#### Work fast

Most female managers said that they needed to work fast, and that they lacked time to carry out tasks and deadlines. For example, they had to be quick and flexible to handle situations that required changes in the team, such as illnesses or additional demands from management. This also applied when they were challenged to find solutions to urgent issues that arose during the course of work. Here, the female managers felt that they had to find a balance in management in being able to be quick, wise and at the same time not to fall short in their own work quality.

Many female managers found that they were less interrupted in their work by others and were thus able to work more efficiently during telework. Most said that the availability of staff became easier during telework, as the ability to get a direct response via a digital chat increased. This saved time searching for colleagues in the office. At the same time, the ability to find quick solutions by daily dialog became more difficult, as the personal physical meetings did not occur during telework, as formulated by R3:

When you do not meet and see each other, you do not become as decisive… or what to say. Procrastination, i.e., postponing decisions… Nothing felt so urgent, because you were not seen. It is also more difficult to make decisions and get a sense of whether you are on the right track. Then once we met, then no matter what we decide, then it went away.

#### Work hard

Most female managers felt that they periodically needed to work hard. This was described as having burdensome periods of high workload, for example, employee interviews, salary periods and follow-up interviews. This required an extra effort from their management:

There must still be time for all the employee meetings, reconciliations you need, certifying time, reports, invoices and all that other stuff too. I have so much more in management, I have a big part which is management. So much booked and in between you have to work. There are many different things that must be submitted. (R6).

In order to work hard, it was important to be more engaged, energizing, calm, stress-resistant and able to push oneself and others, as expressed by R3:

I have to be committed, I have to have energy, I have to take responsibility, I have to push myself and others. I must not stress myself out unnecessarily or get excited. I have to find a good balance… but that’s because I’m so experienced.

The female managers described that the work had not become harder during telework, but it was more difficult to know if you are working with the “right” tasks and if time was used correctly. Given that the number of meetings had intensified, they experienced that their availability to others had decreased. In the words of R7:

It becomes more that you are busy in meetings, i.e., vis-a-vis the team… Maybe I do not feel that working remotely has made it harder. It’s just that it’s a different distribution of time, a different way of working.

#### Excessive work

Most female managers considered themselves to be the “spider in the web,” something that was experienced as both stimulating and exhausting. The stimulating part gave them an opportunity to be involved and to influence. The effort was about handling complex cases in personnel, legal, strategical and organizational matters.

During telework, the female managers did not experience any major differences in planning, but that they had to put a greater commitment in their work-environment responsibility as the employees’ physical and mental well-being varied. However, such mental coaching was experienced as a bit pointless. In the words of R6: “… I do not think you fix that as a manager no matter how you do it. You cannot be the psychologist; you cannot be that mental coach by any means.” The female managers also thought that they were working more during telework. They felt that digital meetings became impersonal, rigid and stilted, which required more commitment from them as the work became more monotonous.

#### Conflicting demands

Most female managers felt that their duties did not directly conflict with each other, as they always had the option of prioritizing and distributing their working day. They often applied their cognitive ability in being solution-focused, which enabled them to do several tasks simultaneously, as R4 expressed it: “Usually it is possible to do two tasks at the same time or to plan so that they go together.”

Conflicts between work tasks were mostly solved, according to the female managers, because everyone in the organization was perceived to have become more efficient during telework. Accordingly, the female managers did not have to monitor different results and outcomes at the same time. This gave them more time to perform better and review the administrative tasks. They prioritized tasks by discussing solutions with their superior manager and colleagues, signaling when they needed support.

Most female managers were involved in projects in addition to their day-to-day duties. Consequently, they needed to plan and prioritize in order to create a reasonable schedule for themselves. The female managers who felt they had time also experienced that they could prioritize better. However, some female managers reported that much of the time was spent prioritizing personnel and operational issues, meaning that their own tasks had to be postponed. In addition, others experienced that much of their time was spent on catching up with conversations that need to be prepared to create a comprehensible structure for the employees. By this, some had to do the remaining tasks in the evenings, early mornings and weekends. In the words of R1:

But I had solved what was most urgent, then I got up early the next day and solved the rest… And if I had not been able to solve it, then I would have told my boss, I will not get this in, it will come tomorrow.

### Control

#### Learn new things

The female managers felt that they learned new things every day in their work, something that they did mainly through their employees, managerial colleagues and their immediate superior manager. New learning occurred when something new or unusual happened at work and in communication with colleagues. They also experienced that they had access to educational programs via the intranet and that the HR department offered telework leadership education. Learning new things was also expressed as:

I also try to read a bit, that is, information emails or things like this that I take part in, look at news sites… it happens daily. New learning platform at the organization, we also have training there… even those “the must trainings” I do. Then there are those that I think I would benefit from, IF I had time. (R6).

However, the female managers lost the “overhearing” during telework, that they got at the office, which reduced learning of new things via their co-workers. But the digital tools facilitated their learning process and opened up for more people to be able to participate in educational training. In the words of R7:

We were not very digital before teleworking at the organization. It has opened up new opportunities… and you have got training and several have been able to participate. It has become easier to be invited to certain briefings that we will still have to hold… This has been easier at a distance.

#### Creativity

Being creative in one’s own work was something that all female managers felt they were given an outlet for, by suggesting new ways of working and finding alternative solutions for their team. This could be expressed in various tasks, for example, in business management, where the female managers were to lead, manage and develop the business in an efficient and legally secure manner, as well as by the correct distribution of resources. They stated that it was by work meetings, discussions and their own opportunity to plan different days of activity for their teams that creativity often occurred.

The opportunity for creativity was, however, hindered during telework. Several of the female managers thought that the physical presence in the office compared to telework contributed to creativity, as they could be more spontaneous based on needs that appeared: “In some way, you become more creative when you are in the office. You pick up something in a different way and you might see something, then you fix it, make it right.” (R5).

Telework could mean, among other things, monotonous information meetings, which were not stimulating enough to awaken creativity:

… it’s more difficult when it’s digital, I think. So if you are in the office or something like that, then you can be creative, now digitally, there have been a lot of PowerPoints… there you can be creative, absolutely, and make things… But no, for me it has been more difficult to be creative in the digital than when it is usually physical. (R7).

#### Variety

All participants said that as a manager they had a great deal of variation in the daily work. They had to work on various issues with a diverse content and subjects such as law, social studies, administration, democracy and values. Tasks could vary between purchasing equipment, paying invoices, booking and planning work-related events, putting up summer or rehabilitation plans. Some of the female managers felt that the personal work tasks (compared to social/personnel issues) were very varied, expressed by R5 as:

… then in the next turn I’m going into a meeting where I’m sitting in a project where we are doing development within the business and then in the next breath I’m suddenly going into a meeting where I’m supposed to have prepared and present a two-hour meeting and the next, then I have some other meeting. So they are very varied.

Telework was experienced to have facilitated unlimited collaboration with employees both within and from other organizations, something that was perceived to provide variety. However, the female managers felt that the tasks became monotonous because they were at the same place, i.e., their home, all the time. In contrast, working in the office was experienced as more varied regarding the physical environment as expressed by R5:

When you are in the office, yes, but then I do not sit in my seat when I have meetings, then you move around more… that way it feels more varied in the office, although it may not really be so.

#### Decision freedom

All female managers said that they had the opportunity to make their own decisions at work, within certain limits from the superior manager. They decided on salaries, rehabilitation measures, recruitment processes and vacations for their employees. It was also the length of service, defined by organization, that clarified for them what to decide upon. They could also decide on the layout for the day, week or month and *how* certain tasks should be delegated. The female managers experienced that they could influence decision making through the weekly reconciliations, together with their superior manager and managerial colleagues: “However, there are many things that we ourselves decide to do. But I always felt that I had the opportunity to influence. I have a great opportunity to be heard there as well.” (R3).

Performing tasks that required presence in the office was, however, perceived as difficult. Here they felt a need to delegate to employees who were present at the office. In the words of R2: “Well, I may have been forced to delegate even more. If I have been sitting at home… maybe I have needed to ask someone else to do it.” (R2).

Freedom to decide was not affected by teleworking, according to most female managers. However, the frequently formal meetings during telework entailed a change by more checking in the chain of decision which led to delays in decision making: “No, the decisions have been made by me. But there are a lot of follow-ups at a detailed level, I have not experienced that before.” (R1).

### Support

#### Superior manager concerned

All female managers felt that superior managers paid attention to and were concerned for the managers’ work. This was done by highlighting and giving suggestions as to what managers needed to improve and what they did well. The feedback was given via coordination, workplace and/or management meetings.

Teleworking had not led to any major change in how the superior managers showed their attention and concern. However, the female managers felt that the workload for the superior managers had increased, and that it was not as easy to ask quick questions and receive direct feedback: “Even if they are not always very good at giving feedback, they do it anyway, in our conversations.” (R5). It was, however, easier to do chat conversations in the office:

You cannot walk by like that if I think that maybe I should tell this to my boss. Then I see that he/she is in meetings all day… Well, then I’ll save this for the reconciliation meeting instead, so it will not be this natural ongoing daily feedback. (R4).

According to some of the female managers, the communication contact had increased during telework, as R3 expressed: “We’ve almost had more contact… Because there has been a lot of focus around this with getting in touch.”

#### Helpful superior manager

All female managers experienced that their superior managers were helpful, as there was mutual dependence in the daily work. Superior managers were helpful by their commitment, weekly reconciliations, support and feedback. The female managers said that they always had the opportunity to contact superior managers when the need arose and that superior managers prioritized being available, providing support during telework, even though their workload had increased. In the words of R5: “… when I need support, he/she is there for me.” and R7: “We have reconciliations, he/she gets in touch, asks how the team is doing… he/she is very committed.”

#### Helpful coworkers

The female managers said that all employees were colleagues. If they had the same type of tasks or were part of a collaboration, they were considered to be close colleagues, facing the same problems, solving them together: “… you have a collaboration with them. We have the same goal, I think, we strive for the same thing and that we depend on each other.” (R6).

Colleagues expressed their support to the female managers by giving tips, praise, and confirmation, as well as expressing concern and sharing their own experiences via suggestions for improvement. In the words of R2: “We have the opportunity to act as a sounding board, it is mutual. They take their time, they are available, offer ideas and suggestions, encourage me when needed, challenge me when needed.”

Willingness to help each other was not affected by telework according to some of the female managers. However, spontaneous help toward colleagues was limited according to some of the female managers. Regarding opportunities for help from co-workers and the social distance to one’s colleagues, R3 said: “The ones you were in the same place as, you have gone away from. Those who you work closely with… you have come closer to. So, the distance has become significantly less important…”

#### Coworkers work together

There was a consensus that colleagues cooperate. All female managers felt that good cooperation was required for the organizational workflow. The cooperation could be expressed in different ways, by asking for help, joint planning and helping to achieve the organization’s results: “We cooperate a lot. We do not work individually; we are a team organization, and we work in a team.” (R3).

According to the female mangers, the cooperation between colleagues was not, in general terms, affected by teleworking. They experienced an advantage of teleworking because digital tools made it easier to get in touch, making collaboration easier. As expressed by R7: “… that’s another forum… it is the change, but not the collaboration itself…”

## Discussion

We investigated female managers’ experiences of occupational demands, control and support in their organizational leadership during telework, as it has been shown that teleworking leadership vs. leadership in-person is associated with different work conditions affecting social interactions, work performance and mental health (Widar et al., 2021; a Vinueza-Cabezas et al., 2022). This is of certain concern for teleworking female managers since they may face different types of work- and family conditions than male managers do (Mann and Holdsworth, 2003; Akram, 2020; Banerjee and Doshi, 2020; Gu et al., 2020; Xie et al., 2021; Rahnfeld et al., 2023).

The present qualitative study was done within a theory-driven approach with three pre-determined main themes of job demand, control and support, each including four predetermined sub-themes, deductively derived from the JDCS-model (Karasek and Theorell, 1990).

Regarding job demands, most of the female managers showed consistent experiences of *work fast, work hard, excessive work, and conflicting demands*. Several aspects of these job demands had not changed during telework while others, like environmental responsibility, work meetings, working more hours, personnel and operational issues, had intensified. The female managers also experienced a difficulty to find quick solutions by daily dialog due to lack of physical meetings during telework.

Work environment responsibility, personnel and operational issues took up a large part of the female managers’ time and placed demands on the work, which meant that they had to prioritize more among their work tasks. This is, in some parts, in accordance with Widar et al. (2021) who found many of the demands experienced by first-line managers to be contradictory. A manager is appointed to be a coach, so it is important to have knowledge of managers’ working conditions to understand their impact on employees. Leading and guiding the learning of others is a big demand for managers. If these managers have little time space and lack support from superior management, it can lead to the managers’ work being perceived as more intense (Billett, 2003). When a manager experiences time demands and does not get the opportunity to control the working time, where all the time is spent solving different problems, his/her space for action is minimized and the individual ends up in a risk of more stress (Langfred and Rockmann, 2016). A manager who is stressed might have difficulties in understanding and handling social situations, such as creating a good team spirit (Braund et al., 2019).

The present results of female managers´ experiences of job demands are of certain concern since women tend to combine several roles in their private sphere and they are expected to carry out chores that belong to the home when working remotely (Mann and Holdsworth, 2003). Also, teleworking women compared to men report more stress and fatigue (Heiden et al., 2021). Female mangers may also be more affected by job demands and psychological distress, and perceive job demands as job-family demands and may therefore be more affected by work–family conflicts and adverse mental health compared to male managers (Akram, 2020; Banerjee and Doshi, 2020; Gu et al., 2020; Xie et al., 2021; Rahnfeld et al., 2023). This, especially if they are teleworking (Mann and Holdsworth, 2003).

Regarding job control, the female managers were shown to have uniform experiences of *learn new things*, *creativity*, var*iety*, and *decision freedom*. This had not changed particularly during telework according to most of the female managers. They were able to manage both their time and working day and had the authority to make their own decisions. This may reduce stress and burnout symptoms and increase mental health, applying the cognitive ability of planning and a solution-oriented way of working (Rafferty et al., 2001; Gao et al., 2021). Self-management in one’s own work situation is a central part of the control at work (Karasek and Theorell, 1990). The female managers expressed that they had control, by being able to learn new things at work via training, communication and educational programs. Therefore, organizations should arrange workarounds, such as training, general knowledge facilitation and follow up of the results (Rafi et al., 2019). Such educational instances may usually help employees to create a better balance in the professional role based on requirements, control and support [see Bakan (2000) and Joo and Grable (2005)]. By this, stronger sustainability may be achieved for both organization and employee (Widar et al., 2022). By offering managers skill development, continuous learning is created. When managers have control over their work situation, their development is stimulated and the motivation to acquire new knowledge increases (Paulsson et al., 2005). The female managers in the present study felt that learning new things via “overhearing” from other colleagues was negatively affected during telework. The “overhearing” can be interpreted as “learning via gossip” which acts as an effective communication channel to spread and share information and encourage learning (Zhu et al., 2021). The female managers were, however, given more coherent time to be able to perform their administrative tasks during telework. An undisturbed work environment gives managers more peace of mind, being more efficient at work. Tasks that require high concentration also require an undisturbed work environment because work performance is affected (Jahncke and Hallman, 2020). Giving the opportunity to try new and alternative work methods may lead to increased creativity (Cai et al., 2019).

Telework enabled more variety by increased collaboration for several of the female managers in the present study, but the work was perceived as less stimulating. By changing the workplace, work tasks- and pace, it is possible to obtain a variation in the work, which is one of the factors for recovery during the working day (Ejlertsson et al., 2018). Managers’ opportunity to recover by varied work tasks may contribute to increased productivity during telework. Being able to influence one’s own working day and how tasks are to be carried out may increase participation and work commitment. This in turn may create a sense of meaning and sustainability in working life (Karasek and Theorell, 1990; McKenna and Jeske, 2021; Korkeakunnas et al., 2023). For female managers, the types of job control experienced by the participants in the present study may spill over to their private life in that they may perceive job control as job-family control (Banerjee and Doshi, 2020; Xie et al., 2021). This may in consequence facilitate their mental health and well-being (Rafferty et al., 2001; Gao et al., 2021).

Regarding social job support, the female managers were shown to have unambiguous experiences of s*uperior manager concerned, helpful superior manager, helpful coworkers,* and *coworkers work together*.

Superior managers were said to express their support by showing interest and commitment and giving feedback, even though the amount of work was perceived to have increased for the superior managers during telework according to some female managers. It is the superior managers who create and shape relationships and collaborations, as they have the main role in developing both the physical and digital organizational culture (Cortellazzo et al., 2019). Receiving support from superiors has a major impact on job satisfaction and can reduce perceived effort at work (Karasek and Theorell, 1990). The female managers in the present study felt that the superior managers paid attention to work performance through good communication and constructive criticism. Support and clarity from superior managers are important components in reducing misunderstandings about the unspoken promises in the “psychological contract” (Shi and Gordon, 2020). When employees show their support, help each other and communicate well with each other, a “collective control” may arise in the organization. This facilitates coping with the demands at work and promotes good health (Johnson, 1989). Obtaining support from superiors and colleagues can minimize work-related stress (Karasek and Theorell, 1990). Also, social support and commitment from a superior manager can reduce the risk of workplace bullying (Goodboy et al., 2017).

Overall, the support was not perceived to have changed during telework. However, some of the female managers in the present study did experience a lack of spontaneous conversation and difficulties in giving and receiving spontaneous help. The latter could be linked to social support at the workplace, through the possibility of helping others, as it increased security and trust among colleagues. According to Meier and Stutzer (2008), individuals who help others may feel appreciated and valuable themselves, and they also report greater well-being. This may reduce the amount of stress and increases further social support [see Curry et al. (2018) for a meta-analysis]. The types of social support experienced by the female managers in the present study may be of certain importance in that such job support may reduce effects of job demands, since women vs. men are more affected by job demands and psychological distress, and perceive job demands as job-family demands (Banerjee and Doshi, 2020; Xie et al., 2021).

The female managers in the present study experienced some of the job demands to have intensified during telework, such as more working, increased number of meetings and that digital meetings require more commitment. Also, some aspects of the job control and social support were experienced as having deteriorated during telework. For example, less “overhearing” and chat conversations in the office, monotonous information meetings and work tasks not stimulating creativity, and less spontaneous help were regarded as consequences of telework. However, telework was reported to have facilitated collaboration within and between organizations. Some aspects of communication, superior manager prioritizing being available and providing support and digital tools made it easier to get in touch with each other. Good social support and self-control reduce the perceived challenges during telework (Wang et al., 2021) which may facilitate well-being and reduce stress. This is of relevance for female managers since the experiences of social support, as with job control, may affect their private life in that they may perceive job support as job-family support (Banerjee and Doshi, 2020; Xie et al., 2021), improving their mental health and well-being (Rafferty et al., 2001; Gao et al., 2021).

In terms of the JDCS model and the transitioning from leadership in-person at the office to telework leadership (Cortellazzo et al., 2019; Terkamo-Moisio et al., 2022), some of the present results indicate the female managers to be in an iso-tense state, an un-healthy state, since they experienced some of the demands to have intensified, while some types of control and support had deteriorated during telework (Karasek and Theorell, 1990). However, at the same time, some types of control and support were said to have increased during telework for the female managers. In general, they also indicated a balance of job demands, control and support during telework and may therefore be in an active state more optimal for their mental health [see Karasek et al. (1998) and Sanne et al. (2005)].

## Limitations and suggestions for future research

Finally, some limitations of the present study should be mentioned: First, we only interviewed one sample of seven female managers from one organization. This might limit the generalizability of the results. It would be of value for future studies to interview different types of participants such as managers and non-managers as well as females and males, from different organizations regarding their experiences of job demands, control and support during telework. Second, one may use a bigger sample in order to draw more elaborated conclusions based on qualitative data such as the present study. Third, the qualitative approach of the present study with seven participants limits the possibilities to generalize the results to a wider population. Therefore, there is a need to quantitatively investigate the phenomena of the present study using a randomized sample technique and a sample big enough to provide sufficient power to the study. Here it would be of value to compare different types of groups such as females vs. males, managers vs. non-managers, teleworker vs. non -teleworkers.

## Conclusion

Female managers face different work- and family conditions when they are teleworking than male managers do. With the aim of investigating female managers’ experiences of job demands, control and support in their organizational leadership during telework and by using a deductive, theory-driven qualitative approach, the present study adds new knowledge to the topic of individual female managers experiences of job characteristics during telework. To our knowledge, the results obtained have not been reported by previous quantitative and /or inductive qualitative studies.

Most of the female managers in the present study might be in the active state based on the demand-control-support model. They experienced several demands, several sources of control, with a high decision-making scope, as well as social support from superiors and coworkers.

Concerning teleworking, organizations might promote sustainable work situation for female managers by facilitating the handling of personnel-, work environmental and operational issues. This might be done by readily available communicational tools, social support, educational programs, variety, less monotonous tasks and meetings and by decision freedom. An individual who has the opportunity to influence his/her work activities, who gets to apply his/her skills in this work relationship, and who has access to support from superior managers and coworkers often experiences reduced stress and increased efficiency. Such a self-directed way of working strengthens the individual’s job satisfaction and increases productivity and sustainability for organizations and employees. For female managers, such experiences of job demands, control and support may have consequences for their family life and affect the balance between their work and family life as well as their mental health and well-being.

## Data availability statement

The data analyzed in this study is subject to the following licenses/restrictions: the material consists of transcribed interviews that may be made avaliable upon reasonable request to the corresponding author. Requests to access these datasets should be directed to ola.nordhall@hig.se.

## Author contributions

ON: Conceptualization, Formal analysis, Methodology, Project administration, Supervision, Writing – original draft, Writing – review & editing. RK: Formal analysis, Investigation, Methodology, Writing – original draft. LT: Formal analysis, Investigation, Methodology, Writing – original draft. IK: Conceptualization, Supervision, Writing – review & editing.
