# Supplementary material for: Female managers’ organizational leadership during telework: experiences of job demands, control and support
Source: Front Psychol. 2024 May 30;15:1335749. doi: 10.3389/fpsyg.2024.1335749 (PMC11171136; doi:10.3389/fpsyg.2024.1335749)
Supplement: Supplementary file 1 [file Presentation_1.pdf]

Appendix 1.

Interview guide

The interview guide below contains the main questions that were asked during all the interviews. Follow-up questions that just were asked in some particular interview/interviews are not part of the interview guide.

Background questions

● How old are you? (age)
● To which gender do you consider yourself to belong?
● What is your education?
● What is your formal position at work?
● How long have you worked in your position?
● How long have you worked within the organization?
● What do your work tasks consist of?

Job Demands

Work fast

● Does your work require you to work quickly?
If Yes: In what way? Can you please describe?
- Has it changed during remote work? How?
If No: What do you think it is due to? Can you please describe?

Work Hard

● Does your work require you to work hard?
If Yes: In what way? Can you please describe?
- Has it changed during remote work? How?
If No: What do you think it is due to? Can you please describe?

Excessive work

● Does your work require a lot of effort?
If Yes: In what way? Can you please describe?
- Has it changed during remote work? How?
If No: What do you think it is due to? Can you please describe?

Conflicting demands/conflicting work tasks

● Do you feel that your work tasks come into conflict with each other?
If Yes: In what way? Can you please describe?
- Have theese demands/ requirements changed during remote work? How?
If No: What do you think it is due to? Can you please describe?

Job Control

Learn new things

● Do you get to learn new things at work?
If Yes: In what way? Can you please describe?
- Has it changed during remote work? How?
If No: What do you think it is due to? Can you please describe?

Opportunity for creativity
● Do you have the opportunity to show your creativity at work?
If Yes: In what way? Can you please describe?
- Has it changed during remote work? How?
If No: What do you think it is due to? Can you please describe?

Variety
● Do you feel that your work is varied?
If Yes: In what way? Can you please describe?
- Do you feel that your work is more varied during remote work? How?
If No: What do you think it is due to? Can you please describe?

Decision freedom
● Do you feel that you have the authority to make own decisions in your work?
If Yes In what way? Can you please describe?
- Has it changed during remote work? How?
If No: What are they due to? Can you please describe?
● Do you have the opportunity to influence your work tasks?
If Yes: In what way? Can you please describe?
- Has it changed during remote work? How?
If No: What do you think it is due to? Can you please describe?

Support
Superior manager concerned
● Does your superior manager show an interest in the work you do?
If Yes: In what way? Can you please describe?
- Has it changed during remote work? How?
If No: What is the reason do you think? Can you please describe?

Helpful superior manager
● Do you find your superior manager to be helpful?
If Yes: In what way? Can you please describe?
- Has it changed during remote work? How?
If No: What do you think is the reason? Can you please describe?

Helpful coworkers
● Who do you consider to be your colleagues?
-Why do you consider them to be your colleagues?
- Do they express their support towards you?
If Yes: How?
If No: What do you think could be the reason?
● Do you feel that your colleagues are helpful?
If Yes: Can you describe in what way?
- Has it changed during remote work? How?
If No: What is it due to? Can you please describe?

Co Workers work together

● Do you feel that you and your colleagues cooperate?

If Yes: Can you describe in what way?

- How has it changed during remote work?

If No: What is it due to? Can you please describe?
